# Identification of a Shiga toxin A‐derived peptide internalized into Gb3 receptor‐bearing cells via interaction with the Shiga toxin B subunit

**DOI:** 10.1002/1873-3468.70403

**Published:** 2026-07-04

**Authors:** Giulia Opassi, João C. Encarnação, Valeria Napolitano, Grzegorz Dubin, Grzegorz Popowicz, Karl Andersson, U. Helena Danielson

**Affiliations:** ^1^ Department of Chemistry for the Life Sciences Uppsala University Sweden; ^2^ Ridgeview Instrument AB Uppsala Sweden; ^3^ Department of Immunology, Pathology and Genetics, Rudbeck Laboratory Uppsala University Sweden; ^4^ Malopolska Centre of Biotechnology, Jagiellonian University Krakow Poland; ^5^ Faculty of Biochemistry, Biophysics and Biotechnology Jagiellonian University Krakow Poland; ^6^ Institute of Structural Biology, Helmholtz Zentrum München Neuherberg Germany; ^7^ Science for Life Laboratory, Uppsala University Sweden; ^8^ Present address: Adamastor Bioservices Srl Cuggiono Italy; ^9^ Present address: Institute of Biostructures and Bioimaging, National Research Council Naples Italy

**Keywords:** biosensor, Gb3 receptors, grating‐coupled interferometry, real‐time cell‐binding assay, Shiga toxin, surface plasmon resonance

## Abstract

Here, we explored the potential of peptides derived from the catalytic A subunit of Shiga toxin (STxA) to be drug carriers. Using time‐resolved biosensor‐based assays we examined the interaction between a variety of STxA peptides (varying in length and end groups) and the cell receptor binding subunit (STxB). Peptides which bound STxB included the C‐terminal α‐helix protruding into the interior of STxB and the ß‐strands binding to its surface. Specifically, the C‐terminal 26‐mer resulted in a stable complex in a physiologically relevant pH range for drug delivery. Real‐time cell‐binding analysis showed that the peptide‐STxB complex binds to and is internalized by Gb3‐overexpressing cancer cell lines with surface‐exposed Gb3 receptors. It highlights STxA‐derived peptides as potential as drug carriers.

## Graphical abstract

The process of internalization of the Shiga toxin A subunit via formation of a complex with the Shiga toxin B subunit, which specifically binds to the Gb3 receptor. The peptide is designed to act as a carrier of drugs into cancer cells.

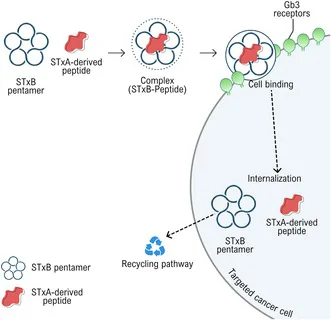

## Abbreviations

**CD**, circular dichroism

**eGFP**, enhanced green fluorescent protein

**FITC**, fluorescein isothiocyanate

**Gb3**, ganglioglobotriaosylceramide

**GCI**, Grating‐coupled interferometry

**SPR**, Surface plasmon resonance

**STxA**, Shiga toxin subunit A

**STxB**, Shiga toxin subunit B

**RT‐CBA**, real‐time cell‐binding assays

Shiga or Shiga‐like toxins (STx) are protein toxins produced by *Shigella dysenteriae* or other bacteria [1, 2]. Their structures belong to the AB_5_ toxin family, in which a monomeric catalytic subunit (STxA) binds to a homopentameric subunit (STxB). The complex binds to extracellular receptors via STxB, whereby it is internalized by endocytosis and reaches the interior of the cell through a complex trafficking process [3]. Early in the process, a furin protease cleaves STxA between a 251 amino acid long N‐terminal toxin moiety (StxA1) and a 41 amino acid long C‐terminal anchoring part (StxA2). However, they remain bound to each other through an intermolecular disulfide bond but become separated in the reducing environment in the ER lumen of host cells. The toxicologically active A1 fragment is thus released into the cytosol, where it inhibits ribosomal protein synthesis.

STxB binds to the ganglioglobotriaosylceramide (Gb3 or CD77) receptor, a glycosphingolipid mainly expressed on the surface of epithelial cells [4]. Since Gb3 is present in certain human tumour cell lines, STxB has been suggested to be a suitable vector for delivery of cancer therapeutics [5]. Research has initially been focused on the design and modification of STxB, using chemical and biotechnological engineering approaches, but also STxA has been engineered and explored for drug delivery [6, 7, 8].

By constructing a fusion protein between enhanced green fluorescent protein (eGFP) and a truncated form of STxA containing the translocation and B subunit association domains, it has been demonstrated that EGFP can be internalized [9]. This concept has later been shown to work also for fusions between the translocation domain from *Pseudomonas aeruginosa* exotoxin A and other proteins [10].

Although the mechanism for internalization and release of the toxic STxA1 fragment into the cytosol is well established [1, 2], it appears that only a small fraction (~4%) of STxA is translocated into the cytosol [11]. Since most of the cell associated STxA seems to be cleaved, forming STxA1 and STxA2, it is apparently due to inefficient reduction of the disulfide bond. More recently, cryo‐EM has revealed a remarkable dynamic behaviour of the STxA subunit in its binding to the pentameric ribosomal P‐stalk—landing platform on the eukaryotic ribosome following the cleavage of STxA [12].

To overcome the complexity of internalization, we were interested in exploring the possibility of using a truncated form of STxA2 as a carrier for intracellular delivery. We therefore focused on designing an STxA2 fragment lacking both the furin cleavage site and the cysteine involved in forming the intermolecular disulfide bond with STxA1. Although the crystal structure of the complex between STxA and STxB has been determined [13], there is no information about the mechanism or kinetics of the interaction or how it is influenced by the different environmental conditions during the trafficking process, such as the acidic environment of endosomes [14]. For this reason, we designed a series of peptides representing truncated STxA. They were based on natural amino acids and the native STxA sequence, but differed in the positions defining the N‐ and C‐terminal residues and end groups (‐NH_2_, acyl, ‐COOH or amide). Their interaction with STxB was studied in a pH range of 5.8 to 7.4, using two different types of time‐resolved biosensor technologies. The interaction of the binders in a real‐time cellular context and their internalization were confirmed via real‐time cell‐binding assays (RT‐CBA).

## Materials and methods

### STxA‐derived peptides

Peptides 1 and 2 were from Peptide Specialty Laboratories, Heidelberg. Peptides 3–8 were purchased from Biomatik Corporation (Ontario, Canada). Lyophilized peptides were re‐suspended in milli‐Q water or 100% DMSO. For LigandTracer experiments, N‐terminally fluorescein isothiocyanate (FITC) labelled Peptide 8 (Peptide 8*) was purchased from Biomatik Corporation (Ontario, Canada). The synthesis was done using FITC‐6‐aminohexanoic acid (Ahx), which includes Ahx as a spacer, improving the stability of the fluorescent label [15]. Lyophilized peptide was re‐suspended in 100% DMSO.

### STxB protein expression and purification

Recombinant STxB (UniProt ID P69179) without the signal peptide (*i.e*. residues 21–89) but with an N‐terminal His‐tag was produced from a pET‐46 plasmid:MAHHHHHHVDDDDKTPDCVTGKVEYTKYNDDDTFTVKVGDKELFTNRWNLQSLLLSAQITGMTVTIKTNACHNGGGFSEVIFR [16]. The plasmid was transformed into 100 μL *E. coli* BL21 (DE3) by heat‐shock and the cells were subsequently plated on solid agarose‐LB medium supplemented with ampicillin. Bacterial colonies were grown in LB medium containing 100 μg·mL^−1^ ampicillin at 37 °C until an OD600 of 0.7 was reached. The system was then cooled down to 18 °C and expression was induced with 1 mm isopropyl β‐D‐thiogalactoside. Harvested cells were lysed by French press (1.4 kbar), the supernatant was filtered and loaded onto a nickel‐chelated agarose affinity column (Qiagen). Elution of the His‐tagged protein was performed in PBS (Medicago AB, Uppsala, Sweden) at pH 8.0, using an increasing concentration of imidazole, as described by the manufacturer [17]. An additional purification step was performed via size exclusion chromatography (SEC) in PBS, using a Superdex 75 Hiload 16/60 column (GE Healthcare/Cytiva, Uppsala, Sweden). Purity was checked by Tris/glycine SDS/PAGE (Bio‐Rad, Hercules, California), and the protein concentration was determined using a NanoDrop ND‐1000 Spectrophotometer (Marshall Scientific, Hampton, New Hampshire).

### Circular dichroism analysis

Peptides were diluted in water to a final concentration of 100 μM. The spectra were recorded in 0.1 nm increments from 190 to 250 nm in a 0.1 cm cuvette, with a bandwidth of 2 nm and a scanning speed of 50 nm·min^−1^, using a JASCO 1.500 instrument. Each spectrum represents the average of three scans. The analysis of the spectra was performed with Spectra Analysis software (Jasco). The Dichroweb online software [18, 19] was used for the deconvolution of the spectra with Contin‐LL [20] and Selcon3 [21] analysis programs.

### Surface plasmon resonance (SPR) biosensor analysis

The interaction between STxA‐derived peptides and STxB was analysed using Biacore 2000 and 3000 instruments (GE Healthcare/Cytiva, Uppsala, Sweden). All experiments were carried out at 25 °C. STxB, at 50 μg·mL^−1^ in 10 mm Na‐acetate at pH 5.0, was immobilized to a level of approx. 2000 RU on a CM5 sensor chip using a standard amine‐coupling procedure [22]. A reference surface, for correction for unspecific binding to the dextran matrix, was created by activating and inactivating a sensor surface via the same procedure (without adding STxB). Immobilization was done in a running buffer composed of PBS at pH 7.4, supplemented with 0.05% Tween20 (PBS‐P^+^). For the interaction analyses, the running buffer was PBS at pH from 5.8 to 7.4, supplemented with 0.05% Tween20 and 1% DMSO.

Peptides were dissolved in the running buffer and diluted to a threefold dilution series, from 10 μm to 41.2 nm. Samples were injected at 50 μL·min^−1^, starting with the lowest concentration. The data were corrected by removing nonspecific signals by subtraction of signals from the reference surface. Solvent correction was also performed, thus compensating for differences in DMSO concentrations between the running buffer and samples. Affinities (K_D_) were estimated by fitting reversible 1:1 binding models, representing 1 or 2 states, to the sensorgrams, using TraceDrawer software (Ridgeview Instruments AB, Uppsala, Sweden).

### Grating‐coupled interferometry (GCI) biosensor analysis

Interaction kinetic analyses between STxB and STxA‐derived peptides were performed at 25 °C using a WAVEdelta system (Creoptix AG, Wädenswil, Switzerland). STxB at 50 μg·mL^−1^ in 10 mm Na‐acetate at pH 5.0 was immobilized via amine coupling in a running buffer 0.2× PBS‐P^+^ at a level of 3000 units, using the same procedure as for SPR‐biosensor experiments (see above). A reference surface was created as above.

Peptides were injected as fivefold increasing concentration series, with a maximum of 1 μm, a flow rate of 50 μL·min^−1^ for 30 s. Four running buffers, consisting of PBS at pH 5.8, 6.4, 6.8 and 7.4, supplemented with 1% DMSO, were used. Six start‐up injections and DMSO solvent corrections were integrated into the experimental design. A buffer exchange procedure was performed to equilibrate the system when a new running buffer was introduced. The total run time for the complete experimental series was 12 h. Kinetic parameters were determined by fitting reversible 1 : 1, one‐step and conformational change models using the WAVEControl software (Creoptix AG, Wädenswil, Switzerland).

### Culture and seeding of cells

Ramos cells (ATCC®CRL‐1596™, RRID: CVCL_0597) and K562 cells (ATCC®CCL‐243™, RRID: CVCL_0004) were cultured in RPMI 1640 (cat. no.: F1215; Merck Sharp & Dohme Ltd., Hoddesdon, UK) supplemented with 10% fetal bovine serum (cat.: no.: F6765; Sigma‐Aldrich; Merck KGaA, Darmstadt, Germany), 2 mm L‐glutamine (cat. no.: K0283; Merck Sharp & Dohme Ltd.), 100 IU penicillin and 100 μg·mL^−1^ streptomycin (cat. no.: A2213; Merck Sharp & Dohme Ltd.). All experiments were performed using cells that were routinely tested and confirmed to be free of mycoplasma contamination. The cells were acquired from ATCC and used in the first year of expansion, with the project conducted over 3 months. The cells were sent for authentication using short tandem repeat (STR) profiling (Eurofins Genomics, 85 560 Ebersberg, Germany).

For easy comparison between the different RT‐CBA with STxB‐Peptide 8* to living cells, ligands were measured simultaneously using LigandTracer MultiDish 2×2 for coatings (cat. no.: 1‐4‐204; Ridgeview Instruments AB, Uppsala, Sweden). MultiDish 2×2 was coated with a Biocompatible Anchor for cell Membranes—BAM (SUNBRIGHT® OE‐040CS; NOF Corporation) for Ramos and K562 cells since the improvement of cell attachment was needed. Cell seeding to the MultiDish was performed according to the manufacturer's instructions.

### Real‐time cell‐binding assays

The interactions of Peptide 8* and the STxB‐Peptide 8* complex with Ramos cells were measured in a RT‐CBA with LigandTracer® Green (Ridgeview Instruments AB), using a blue (488 nm)—green (535 nm) detector. Measurements were conducted at room temperature in CO_2_ Independent Medium (18 045 088; Gibco™, Thermo Fisher Scientific) and started with a short baseline measurement in the absence of labelled complex. The STxB‐Peptide 8* complex was preincubated at 4°C for 1 h. After 1 h, the complex was added stepwise to the medium to give a final concentration of 30 nm and 90 nm of STxB, and 411 nm and 1233 nm of Peptide 8*. Association phase data were subsequently recorded for a defined time (typically 3–6 h). The solution was then replaced with fresh medium to monitor the dissociation of the STxB‐Peptide 8* complex from the cells. For the long association experiment, STxB was added to a final concentration of 60 nm and Peptide 8* to a final concentration of 822 nm. Data analysis RT‐CBA traces produced with LigandTracer Green were analysed using the evaluation software TRACEDRAWER 1.8 (Ridgeview Instruments AB). Data were normalized to allow a simple comparison of binding curves.

## Results

### Design of STxA‐derived peptides

Based on the crystal structure of the STxA‐STxB complex [13], peptides representing the C terminus of STxA were designed to explore the capability of truncated STxA to interact with STxB (Fig. 1, Table 1). Since the crystal structure lacks density for the last six residues of the C terminus (…RRTISS), the importance of residues beyond the structurally ordered α‐helix (red in Fig. 1) was analysed. Similarly, the importance of the presence or completeness of the ß‐sheet (green in Fig. 1) preceding the α‐helix was investigated. Also, the effect of modified end groups on the binding was examined, specifically the importance of the native negative charge (‐COO^−^) at the C terminus. All peptides had a sequence corresponding to the native STxA sequence and contained only natural amino acids.

**Fig. 1:**
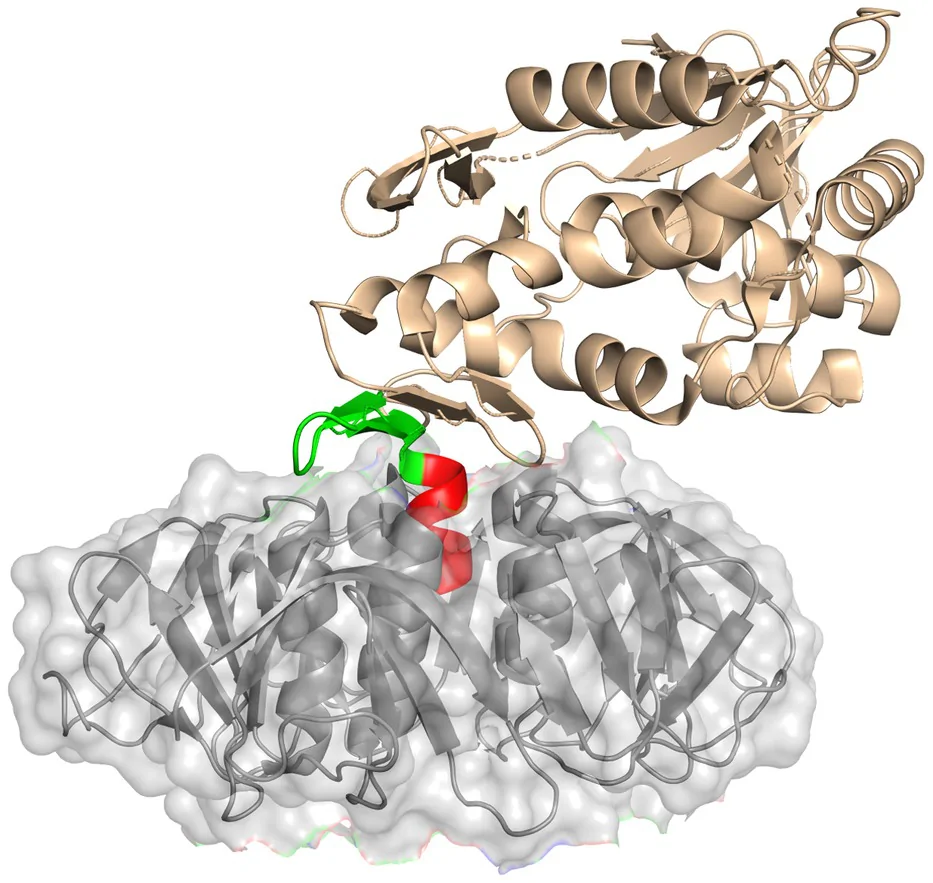
Crystal structure of the STxA and STxB complex (1DM0.pdb), with the C‐terminal region of STxA (top) with its C‐terminal α‐helix (red) inserted into the pocket of the STxB pentamer (grey, bottom) and a ß‐sheet (green) at the STxB surface). The image was made using the PyMOL Molecular Graphics System, Version 3.0 Schrödinger, LLC.

**Table 1:** Sequences and structural features of STxA‐derived peptides, and their capability to interact with STxB, as detected by biosensor analysis (Figs 3 and 4). Residues annotated as part of a C‐terminal ß‐sheet and α‐helix in the crystal structure of STxA (Fig. 1) are highlighted (underlined and bold, respectively). The N‐ and C‐terminal end groups are specified.

Peptide	Sequence	N‐term. Group	C‐term. Group	STxB interaction detected
STxA_288‐315_ ^a^	‐RVRGITHNKILWD **SSTLGAILMRRTISS**	–^a^	COOH	–^a^
1	**TLGAILMRRT**	NH_2_	COOH	No
2	RVRGITHNKILWD **SSTLGAILMRRT**	NH_2_	COOH	Yes
3	D **SSTLGAILMRR**	Acyl	Amide	No
4	D **SSTLGAILMRR**	NH_2_	Amide	No
5	D **SSTLGAILMRRTISS**	Acyl	COOH	No
6	D **SSTLGAILMRRTISS**	NH_2_	COOH	No
7	RGITHNKILWD **SSTLGAILMRR**	NH_2_	Amide	Yes
8	RGITHNKILWD **SSTLGAILMRRTISS**	NH_2_	COOH	Yes

^a^ This is the native C‐terminal region of the protein.

As a start, Peptides 1 and 2 were designed (Table 1). They lacked the three most C‐terminal residues (ISS) which were assumed to contribute little to binding as they were not resolved in the crystal structure. Based on pilot experiments (see below), six additional peptides were designed to further explore the critical features of the interaction. Peptides 5, 6 and 8 include the complete/native C terminus while Peptides 3, 4 and 7 lack the four most C‐terminal residues (TISS) and Peptides 2, 7 and 8 have the complete ß‐sheet.

### Secondary structure analysis of STxA‐derived peptides

The STxA‐derived Peptides 3 to 8 were analysed by circular dichroism (CD). The resulting spectra were subsequently subjected to deconvolution analysis to quantitatively assess the presence of detectable secondary structure (Fig. 2).

**Fig. 2:**
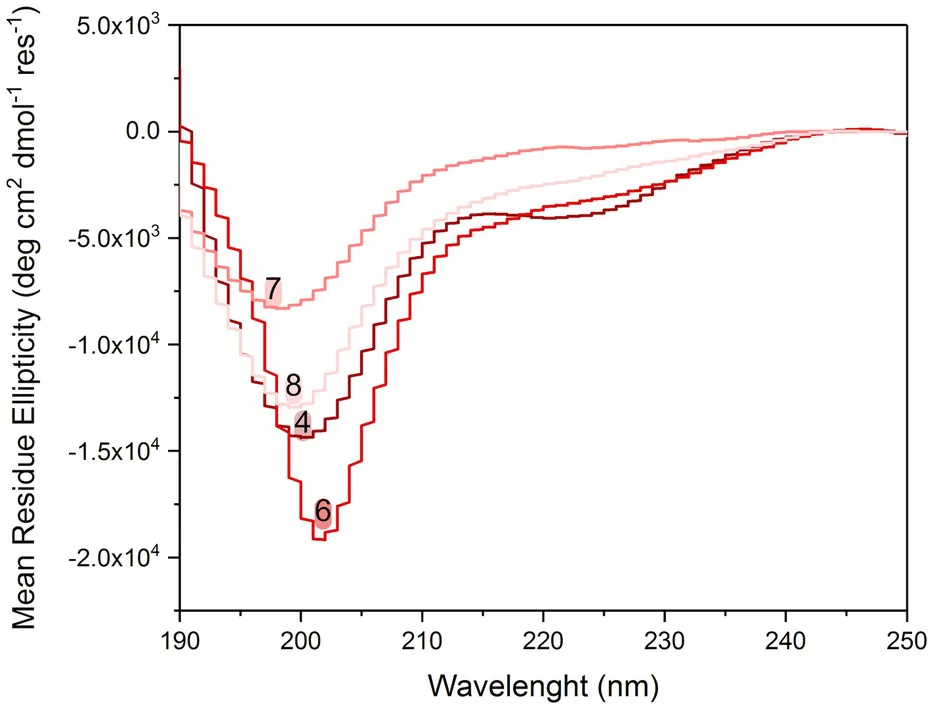
Circular dichroism spectra of STxA‐derived Peptides 4, 6, 7 and 8. The peptides were diluted in water to 100 μm to avoid signal interference due to buffer absorption. Spectra for Peptides 3 and 5 are not shown since their sequences and spectra are identical to those for Peptides 4 and 6, respectively.

The far‐UV CD spectra of Peptides 3 and 4 exhibited a pronounced negative band at 201 nm and a weak band at 222 nm, a spectral signature characteristic of predominantly disordered conformations. Deconvolution of the spectra suggests that these peptides contain only a minor α‐helix content (~20%). Similarly, the spectra of Peptides 5 and 6 displayed a strong negative band at 200 nm, consistent with a disordered backbone conformation. However, the presence of a broad negative region spanning 208–215 nm indicates a more pronounced contribution from α‐helical structure (~50%), according to the deconvolution. Finally, analysis of Peptides 7 and 8 spectra suggests the presence of additional structural features indicative of a greater conformational heterogeneity. Spectral analysis suggests the presence of α‐helical (~20%), β‐strand (~15%) and turn (~20%) components, with the remaining fraction corresponding to unordered conformations.

### Kinetic analysis of STxA peptide—STxB Interactions

The interaction between STxA‐derived peptides and STxB was analysed by two types of biosensor technologies. Initially, a surface plasmon resonance (SPR) biosensor‐based assay was developed by immobilizing STxB to a sensor chip by amine coupling. The first two peptides designed, Peptide 1 and Peptide 2, were injected over the sensor surface in a concentration series up to 2 μm. An interaction was not detected with Peptide 1, but it was for Peptide 2 (Table 1, data not shown). This confirmed that STxB was functional after immobilization and that the developed assay had the sensitivity and specificity to analyse the interactions of interest. Moreover, the results revealed that a truncated C‐terminal fragment of STxA could form a complex with STxB, but that an STxA‐derived peptide without a ß‐sheet and a truncated α‐helix did not have the critical structural elements for a detectable interaction with STxB.

The second series of designed peptides (Peptides 3–8) were analysed with the SPR‐biosensor assay in a series of independent experiments, using new surfaces prepared specifically for the analysis. At all conditions explored, only the longest peptides (7 and 8) were detected to interact with STxB (Table 1). As an alternative, and potentially more sensitive approach, a grating‐coupled interferometry (GCI) biosensor assay was developed and used to analyse the peptides. A matched experiment, using the same concentration series as in the SPR‐biosensor analysis, resulted in similar detection and kinetic profiles, confirming only Peptides 7 and 8 are able to interact with immobilized STxB. Further experiments were therefore focused on understanding the kinetic details of the interactions and the effect of pH. Peptides were analysed in concentration series at pH 7.4, 6.8, 6.4 and 5.8, using both the SPR and GCI biosensor assays.

The two methods gave consistent data, with only the longest peptides (Peptides 7 and 8) interacting at all pH values used (Fig. 3 and Table 2, and Fig. 4 and Table 3, respectively). Moreover, a visual inspection revealed that the peptides had different kinetic profiles, with the observed association and dissociation rates being faster for Peptide 7 than Peptide 8.

**Fig. 3:**
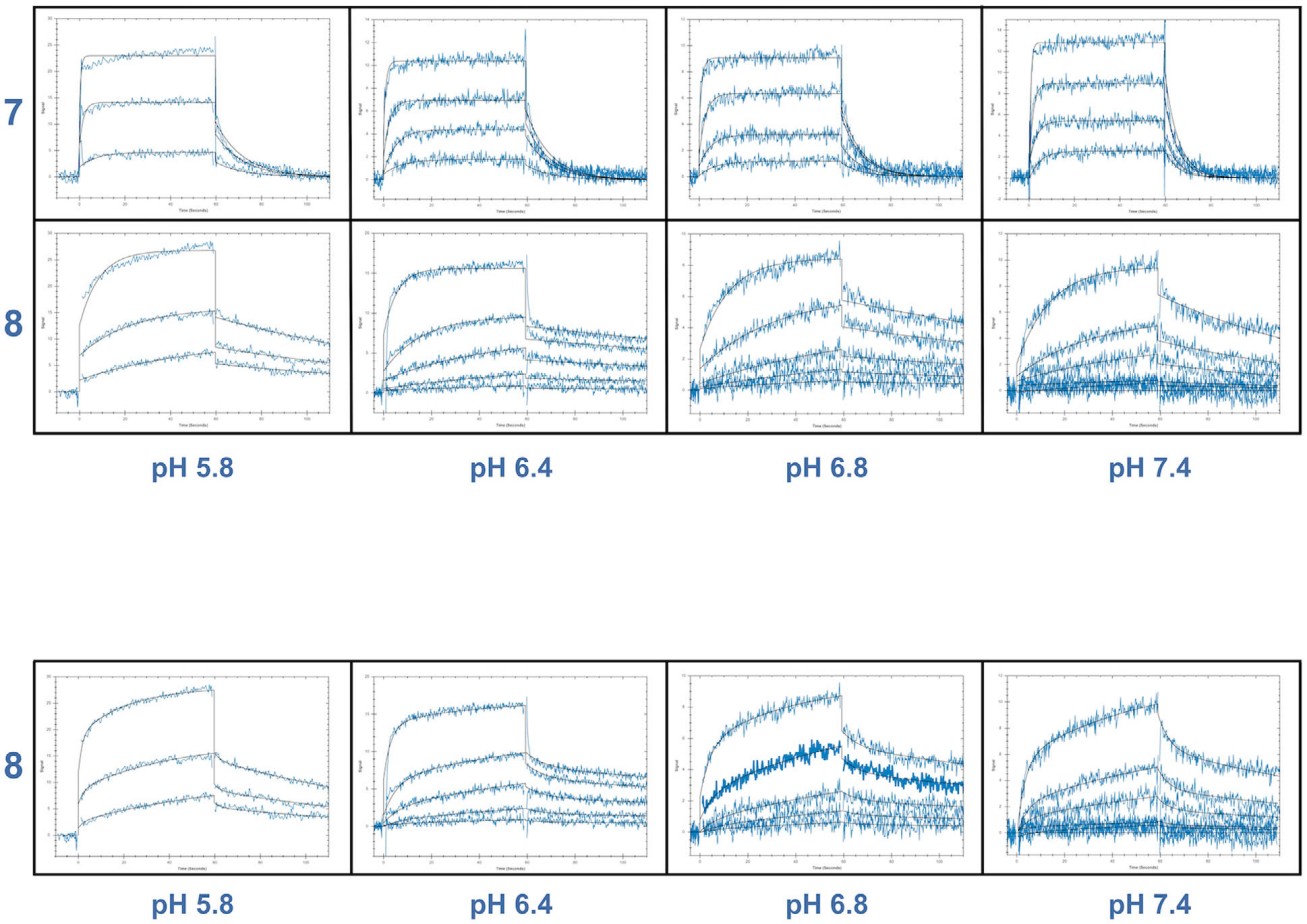
SPR‐biosensor analysis of STxA‐derived Peptides 7 and 8. Sensorgrams of peptides injected in a threefold concentration series up to 10 μm, represented as Signals (RU) over Time (s). Overlays (black lines) represent theoretical curves from fitting data with 1:1 binding (top) and 1:1 Two State (bottom) models. The estimated kinetic parameters are presented in Table 2.

**Table 2:** Interaction parameters and assay data estimated from the fitting in Fig. 3.

			pH			
	Model	Constants	5.8	6.4	6.8	7.4
Peptide 7	1:1	*k*_*a*_ *(*M^−1^s^−1^)	4.38×10^5^	8.01×10^5^	6.82×10^5^	3.95×10^5^
		*k*_*d*_ *(*s^−1^)	8.2×10^−2^	9.85×10^−2^	1.20×10^−1^	1.70×10^−1^
		K_D_ (nM)	187	123	177	431
Peptide 8	1:1	*k*_*a*_ *(*M^−1^s^−1^)	1.07×10^4^	1.77×10^4^	2.34×10^4^	1.82×10^4^
		*k*_*d*_ *(*s^−1^)	8.63×10^−3^	4.09×10^−3^	5.59×10^−3^	1.18×10^−2^
		K_D_ (nM)	804	231	238	646
	1:1 Two state model	*k*_*a1*_ *(*M^−1^s^−1^)	2.58×10^4^	2.31×10^4^	3.18×10^4^	3.14×10^4^
		*k*_*d1*_ *(*s^−1^)	3.05×10^−1^	9.66×10^−2^	1.07×10^−1^	1.86×10^−1^
		K_D1_ (nM)	1590	652	499	1710
		*k*_*2*_ *(*s^−1^)	8.41×10^−2^	2.98×10^−2^	4.22×10^−2^	3.19×10^−2^
		*k*_*‐2*_ *(*s^−1^)	1.13×10^−2^	4.63×10^−3^	6.27×10^−3^	9.19×10^−3^

**Fig. 4:**
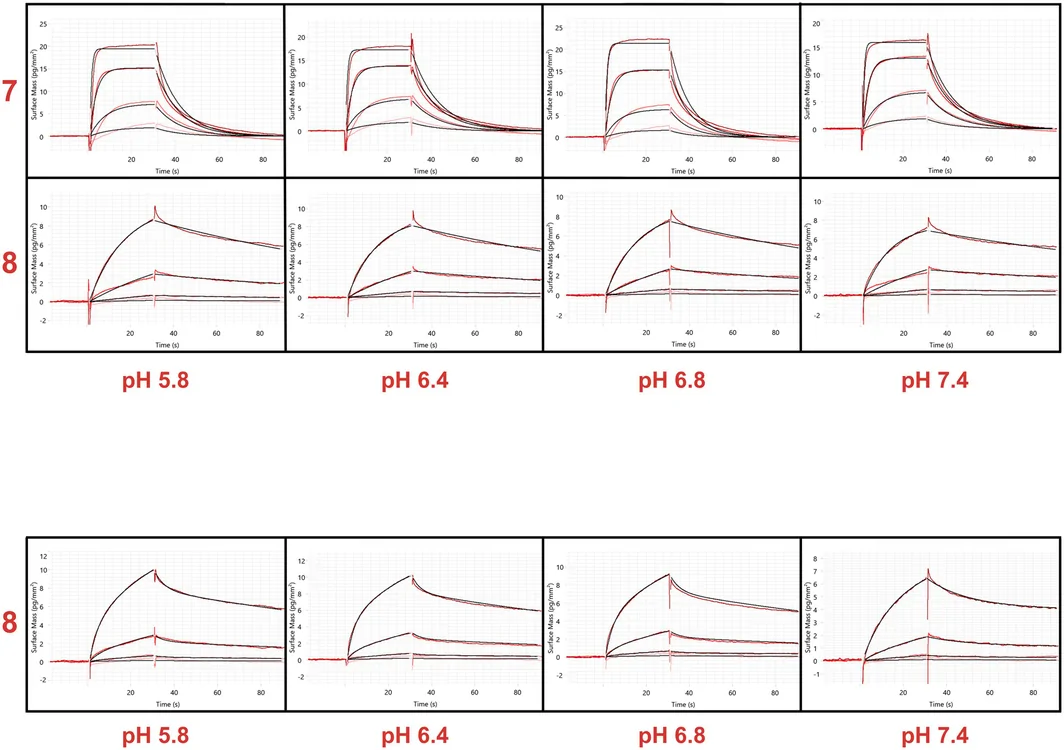
GCI biosensor analysis of STxA‐derived Peptides 7 and 8. Sensorgrams of peptides injected in fivefold concentration series up to 1 μm, represented as Surface Mass (pg·mm^2^) over Time (s). Overlays (black lines) represent theoretical curves from fitting data with 1:1 binding (top) and Conformational Change (bottom) models. The estimated kinetic parameters are presented in Table 3.

**Table 3:** Interaction parameters and assay data estimated from the fitting in Fig. 4.

			pH			
	Model	Constants	5.8	6.4	6.8	7.4
Peptide 7	1:1	*k*_*a*_ *(*M^−1^s^−1^)	1.20×10^6^	1.22×10^6^	1.01×10^6^	1.40×10^6^
		*k*_*d*_ *(*s^−1^)	9.18×10^−2^	8.27×10^−2^	1.11×10^−1^	8.36×10^−2^
		K_D_ (nM)	76.6	67.5	109.5	59.8
Peptide 8	1:1	*k*_*a*_ *(*M^−1^s^−1^)	5.56×10^4^	6.62×10^4^	6.05×10^4^	7.75×10^4^
		*k*_*d*_ *(*s^−1^)	7.41×10^−3^	7.38×10^−3^	7.51×10^−3^	5.75×10^−3^
		K_D_ (nM)	133.3	111.5	124.1	74.1
	1:1 Two state model	*k*_*a1*_ *(*M^−1^s^−1^)	6.60×10^4^	1.05×10^5^	8.99×10^4^	4.20×10^4^
		*k*_*d1*_ *(*s^−1^)	1.46×10^−1^	1.93×10^−1^	1.58×10^−1^	2.91×10^−2^
		K_D1_ (nM)	2212	1838	1758	693
		*k*_*2*_ *(*s^−1^)	6.77×10^−2^	7.52×10^−2^	6.83×10^−2^	2.39×10^−2^
		*k*_*‐2*_ *(*s^−1^)	8.26×10^−3^	8.00×10^−3^	8.47×10^−3^	1.11×10^−3^

Mathematical analyses of the sensorgrams revealed that Peptide 7 was well described by a reversible 1:1 model consisting of a single step, whereas this model was not adequate for Peptide 8, which showed a more complex interaction. It was well described by a model that included an additional reversible step after an initial encounter complex has been formed, representing a conformational change/induced fit model. The analysis showed that Peptide 7 had a slightly higher affinity than Peptide 8 due to a faster association rate constant (*k*_a_), although the dissociation rate constant (*k*_d_) was also faster. This is hypothesized to be a consequence of the flexibility of the four C‐terminal residues only present in Peptide 8.

### Real‐time cell‐binding analysis (RT‐CBA) of the Peptide 8–STxB Complex

Peptide 8 was selected for cell studies since it formed the most stable complex with STxB, as compared with Peptide 7. This is seen as a slower dissociation rate in Figs 3 and 4 (Tables 2 and 3). FITC fluorophore was attached to the N‐terminus (Peptide 8*), to enable the detection of the interaction in the fluorescence‐based RT‐CBA. The stepwise addition of the STxB‐Peptide 8* complex to the cells resulted in a biphasic association, with an initial rapid signal increase, followed by a slower linear increase (Fig. 5A). In the dissociation phase, there was an initial rapid release of 65–70% of the labelled complex during the first hour, but approximately 24% of the complex remained in the cells after 7 h.

**Fig. 5:**
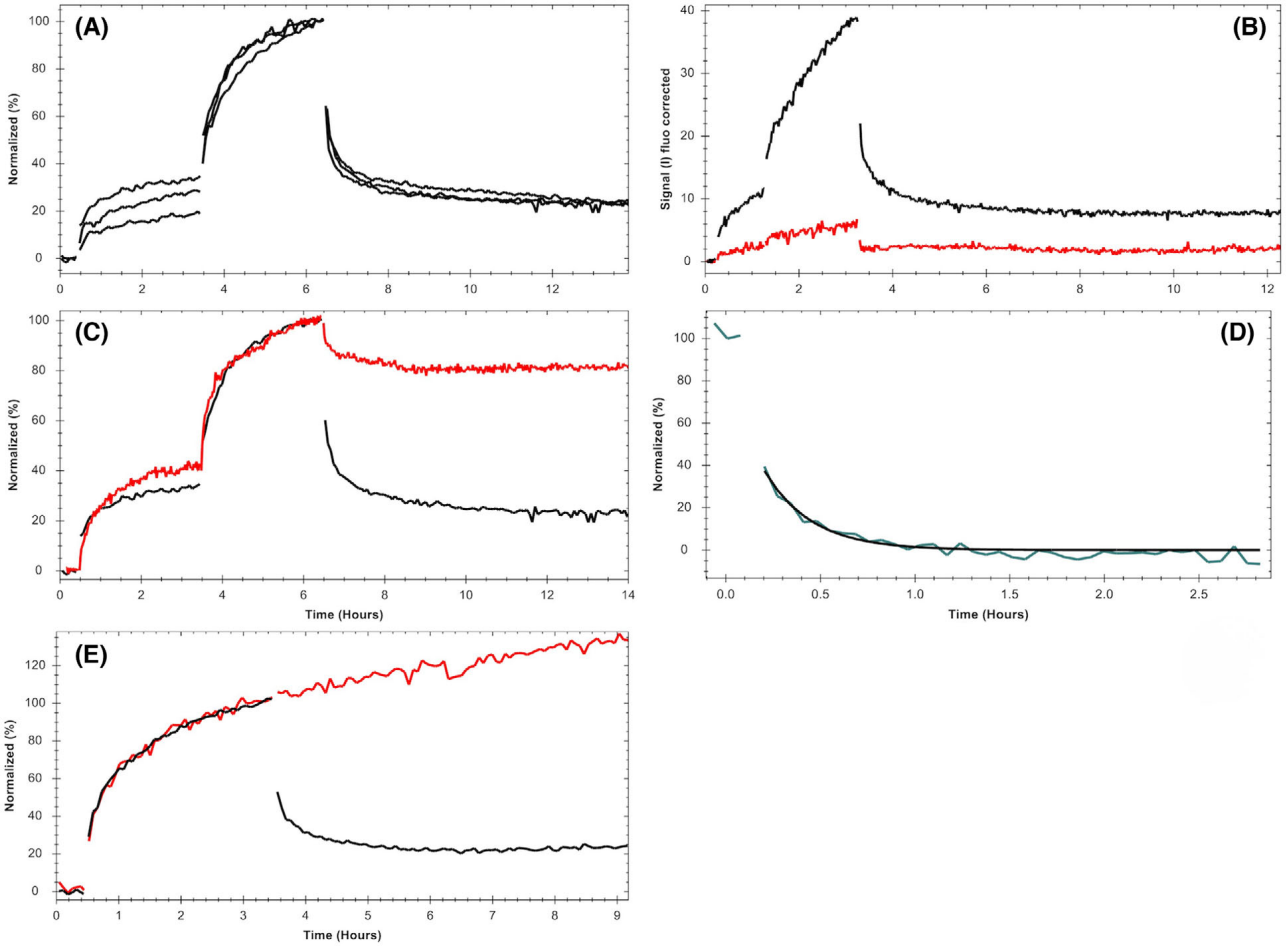
Evaluation of binding and internalization of labelled STxB (STxB‐FITC) and STxB‐Peptide 8* in complex to live Ramos cells via RT‐CBA and confocal microscopy. RT‐CBA binding curves for (A) STxB‐Peptide 8* added to cells using 30 and 90 nm STxB (*n* = 3). (B) Peptide 8* in the presence (black curve) and absence (red curve) of STxB. (C) STxB labelled with FITC (red) and STxB‐Peptide 8* (black). (D) Curve generated by subtracting the dissociation signal of STxB‐Peptide 8* curve from the STxB labelled with FITC curve, and (E) STxB‐Peptide 8* incubated with a final concentration of 60 nm over a long incubation time (>3 h, red), and short incubation times (3 h—black).

A control experiment was performed with Peptide 8* alone, confirming that the peptide only binds to the Ramos cells in the presence of STxB (Fig. 5B black). The low signal for the peptide alone is probably due to unspecific binding to Ramos cells (Fig. 5B red).

To get more information about the association phase of the STxB‐Peptide 8* complex, it was compared with binding of STxB directly labelled with FITC. The association phase of STxB‐Peptide 8* and STxB‐FITC to living cells was characterized by similar parameters (Fig. 5C), whereas the dissociation was faster for STxB‐Peptide 8 at the first hour compared with STxB‐FITC. This faster dissociation could reflect the peptide dissociation from STxB or dissociation of STxB‐Peptide 8* from the cell surface. Moreover, assuming FITC does not significantly impact the binding of STxB with the cell membrane, it is possible to estimate the dissociation rate of the Peptide 8* from STxB in living cells by subtracting the dissociation signal from STxB‐FITC and STxB‐Peptide 8* (Fig. 5D). The resulting subtracted data allowed calculation of a dissociation rate from the Peptide 8* to STxB in a living environment at 1.12 × 10^−3^ s^−1^.

For understanding the impact of time on the binding of STxB‐Peptide 8*, an experiment without dissociation was performed (Fig. 5E, red). As observed in a recent study with other STxB constructs [16], prolonging the incubation time allows one to obtain more information on the slower continuous process. The biphasic behaviour showed had an initial quick binding event, followed by a linear phase being constant for more than 10 h, indicating that there are (at least) two processes present reflected on the binding curve, with a first rapid event being followed by a slower continuous process.

The interaction data suggest a direct interaction between the STxB‐Peptide 8* complex and Gb3 receptors, dissociation of Peptide 8* from STxB or of the STxB‐Peptide 8* complex from the cells, and internalization of the STxB‐Peptide 8* complex. This binding behaviour and internalization from STxB was also shown in a previous study [16].

## Discussion

Initial experiments confirmed that a C‐terminal fragment of STxA was sufficient for interaction with STxB, and that the developed SPR‐biosensor assay was able to detect the interaction. Since a fragment had to contain more than just part of the C‐terminal α‐helix (Peptide 1) to bind, and the longer peptide that was confirmed to bind (Peptide 2) contained additional structural features, the experiments gave the basis for a rational design of shorter peptides that revealed the region required for binding. Peptide 2 was useful as a positive control. The interaction was explored over a range of pH since it has been demonstrated that the mechanism of StxB‐StxA internalization involves a lengthy trafficking process via different organelles, such as early endosomes (pH 5.8–6.8) and the Golgi apparatus (pH ca. 6.5), before reaching the endoplasmatic reticulum (pH 7.2).

A comparison of the interaction data for Peptides 7 and 6 revealed that the four very C‐terminal residues in native STxA (…TISS) influenced the interaction with STxB, even if the crystal structure of the complex lacks density for the last six residues. The faster interaction kinetics (higher *k*_a_ and *k*_d_ values) for Peptide 7 can thus be due to the absence of these flexible C‐terminal residues. Their presence in Peptide 8 thus appeared to slow down the association and dissociation rates, resulting in the formation of a significantly more stable complex with STxB. In addition, the complexity of the interaction was increased, indicating that the interaction mechanism involved multiple conformations. The presence of the ß‐sheet preceding the α‐helix was also found to be critical for binding; none of the peptides without the ß‐sheet could form a detectable complex with STxB. Finally, no obvious pH trends were observed in the kinetic rates of the two peptides studied in detail, although there was a detectable variation.

Two biosensor technologies were used to characterize the binding interaction between immobilized STxB and the peptides: SPR and GCI. Both sensor surfaces were set up in the same way and essentially the same experimental conditions were used. The results were qualitatively comparable, but absolute numbers for the kinetic parameters differed. This is not unusual and does not constitute an issue for this study where analysis was focused on differences between peptide analogues and not determination of universal interaction kinetic parameters. Further controls would be necessary in order to determine whether the differences between the two methods are a consequence of different detection or details of how the experiments were carried out and analysed.

A recent RT‐CBA based study revealed new features of STxB binding and cell internalization by studying the effects of temperature, concentration and time [16]. The same method was used herein to understand the capacity of STxB to carry Peptide 8 into living cells. When combining data from concentration series of STxB‐Peptide 8* (two consecutive steps to final concentrations of 30 nm and 90 nm of STxB, and 411 nm and 1233 nm of Peptide 8*), it was evident that the binding dynamics of STxB with cellular receptors cannot be described by a single, one‐step interaction. The complexity of STxB binding to living cells is described in more detail in the study by Encarnação and collaborators [16]. The biphasic binding curves observed for the interaction between the STxB‐Peptide 8* complex and STxB had an initial fast binding event followed by a slow linear increase. Since steady‐state was not reached, it could possibly represent an internalization/receptor recycling mechanism. Studies with Peptide 8* alone, that is, without STxB being present, showed some unspecific binding. However, in the presence of STxB, the signal from the STxB‐Peptide 8* complex interaction with the cells were significantly higher. Moreover, the dissociation of Peptide 8* from cells was almost complete in the absence of STxB while in the presence of STxB, Peptide 8* was found as a complex with STxB inside the cells. However, a dissociation of Peptide 8* from STxB was observed during the first hours of the experiment. This signal decrease is hypothesized to be due dissociation of Peptide 8* from STxB bound to the cell membrane, while the remaining signal represents Peptide 8* that has been internalized into the intracellular environment alone or complexed with STxB.

## Conclusions

This first study of the interaction kinetics between STxA peptides and STxB has revealed the critical C‐terminal residues of STxA involved in its binding to the pentameric receptor binding subunit STxB and has highlighted features of the mechanism involved. Cells studies have confirmed that a complex of peptides representing the C‐terminal region of STxA can specifically interact with Gb3‐overexpressing cell lines in the presence of STxB and be internalized. These findings suggest that a short peptide could potentially serve as a therapeutic linker for targeting Gb3‐overexpressing cell lines through a ‘molecular‐shuttle’ system. The peptide's theoretical advantage lies in exploiting the natural STxA‐STxB interaction while potentially enabling a novel delivery mechanism. Unlike full‐length STxA or engineered STxB variants that form irreversible complexes requiring proteolytic cleavage for drug release, the short peptide identified appears to have sufficient affinity for STxB allowing cellular uptake while allowing spontaneous intracellular dissociation inside cells. This hypothetical context‐dependent release would leverage the natural binding equilibrium: The peptide‐STxB interaction might be stable enough for extracellular transport yet readily reversible under intracellular conditions, potentially eliminating the need for engineered cleavage sites or external triggers. Such a mechanism could enable STxB carrier recycling for subsequent transport cycles, theoretically improving delivery efficiency compared with traditional ‘use‐and‐discard’ conjugate systems. However, demonstrating this recyclable carrier concept requires further studies examining intracellular release mechanisms, carrier fate postdelivery and overall therapeutic efficacy compared with conventional approaches.

## Conflicts of interest

Ridgeview Instruments AB (RIAB) develops and sells the device LigandTracer, which is described in the manuscript. João Encarnação and Karl Andersson were employed by RIAB when the experiments were performed. Karl Andersson is a shareholder of RIAB. RIAB acknowledges the adherence to all FEBS Letters policies on sharing data and materials. All equipment described in the report is commercially available, and no patents restrict the use of the described assays. Valeria Napolitano, Giulia Opassi, Helena Danielson, Grzegorz Dubin and Grzegorz Popowicz declare that they have no conflicts of interest to disclose.

## Author contributions

GO, JCE and VN designed all the experiments. GO, JCE and VN performed all the experiments. GO and JCE wrote the manuscript with support from UHD. GP designed the expression plasmids. GD, UHD and KA provided crucial input and review on methodology. All authors discussed the results and commented on the manuscript.

## Data Availability

The data that support the findings of this study are available from the corresponding author upon reasonable request.
